# Plant-Produced Broadly Neutralizing Influenza Monoclonal Antibody CR9114 Exhibits Activity Against Heterologous Avian Influenza Viruses

**DOI:** 10.3390/vaccines14030222

**Published:** 2026-02-28

**Authors:** Janejira Jaratsittisin, Win Win Aung, Kaewta Rattanapisit, Pipob Suwanchaikasem, Wayu Matphong, Kanjana Srisutthisamphan, Nanchaya Wanasen, Samaporn Teeravechyan, Waranyoo Phoolcharoen

**Affiliations:** 1 Center of Excellence in Plant-produced Pharmaceuticals, Chulalongkorn University, Bangkok 10330, Thailand; janejira.j@chula.ac.th (J.J.); 6773009833@student.chula.ac.th (W.W.A.); 2 Department of Pharmacognosy and Pharmaceutical Botany, Faculty of Pharmaceutical Sciences, Chulalongkorn University, Bangkok 10330, Thailand; 3 Baiya Phytopharm Co., Ltd., Bangkok 10330, Thailand; kaewta.r@baiyaphytopharm.com (K.R.); pipob.s@baiyaphytopharm.com (P.S.); 4 Virology and Vaccine Technology Research Team, National Center for Genetic Engineering and Biotechnology (BIOTEC), National Science and Technology Development Agency (NSTDA), Pathum Thani 12120, Thailand; wayu.mat@ncr.nstda.or.th (W.M.); kanjana.sri@biotec.or.th (K.S.); nanchaya.wan@biotec.or.th (N.W.); samaporn.tee@biotec.or.th (S.T.)

**Keywords:** universal influenza, monoclonal antibody, avian influenza, *Nicotiana benthamiana*

## Abstract

**Background:** The continual emergence of antigenically drifted avian influenza viruses poses a persistent threat to global health and underscores the need for broadly protective approaches. Stem-directed monoclonal antibodies targeting conserved hemagglutinin (HA) epitopes are a promising strategy to address viral diversity. CR9114 is a broadly neutralizing antibody previously reported to recognize a conserved HA stem region across influenza A and B viruses. **Methods:** CR9114 was transiently expressed in *Nicotiana benthamiana* and characterized for protein integrity, assembly, and glycosylation. Binding to recombinant hemagglutinin was assessed, and neutralizing activity was evaluated against antigenically distinct avian influenza A viruses using in vitro neutralization assays. **Results:** Plant-produced CR9114 was correctly assembled as a human IgG1 κ antibody and displayed a high-mannose glycosylation profile. The antibody showed strong binding to recombinant H5 hemagglutinin (Kd = 0.15 µg/mL) and potently neutralized recent avian influenza isolates, namely A/Jiangsu/NJ210/2023 (H5N1; NT_50_ = 1589) and A/Gansu/23277/2019 (H7N9; NT_50_ = 177), demonstrating cross-subtype neutralization despite known glycan-associated constraints in Group 2 viruses. **Conclusions:** These findings demonstrate that *N. benthamiana* is a viable platform for the rapid production of functional broadly neutralizing anti-influenza antibodies. The preserved activity of plant-produced CR9114 against contemporary avian influenza strains supports its continued evaluation as a broadly protective therapeutic candidate and highlights the potential of plant molecular pharming approaches to contribute to pandemic preparedness.

## 1. Introduction

Influenza remains a critical global threat, causing up to 650,000 deaths annually and carrying an economic burden exceeding US$100 billion [1,2]. While seasonal vaccines require annual updates, the escalating H5 avian influenza crisis in 2025 has highlighted the urgent need for “universal” interventions. Global health security faced new challenges in late 2025 with the first-ever human infection and subsequent death from influenza A(H5N5) in the USA, marking a significant expansion of H5 subtypes infecting humans [3]. Simultaneously, Cambodia experienced a sharp resurgence of human A(H5N1) cases, with 17 infections and nine deaths reported by November 2025, nearly triple the previous year’s total, linked to a highly virulent novel reassortant [4]. These evolving threats underscore the limitations of strain-specific vaccines and emphasize the importance of broadly neutralizing antibodies (bnAbs) like CR9114. By targeting conserved regions of the hemagglutinin (HA) stem, such interventions provide essential cross-protection against diverse and emerging highly pathogenic strains that bypass conventional seasonal immunity.

Broadly neutralizing antibodies targeting the conserved HA stem have emerged as powerful candidates for universal influenza therapy, beginning with early group 1 restricted antibodies such as CR6261, F10 and CR6020, which block membrane fusion by engaging the hydrophobic groove between HA1 and helix A [5]. The first major breakthrough in achieving true cross-group activity was FI6v3, which exhibited binding to all 16 HA subtypes in vitro and protected mice prophylactically and therapeutically from lethal H1, H3 and H5 infections [6]. A more advanced universal solution is CR9114, a VH1-69–derived antibody that not only neutralizes diverse group 1 and group 2 influenza A viruses but also uniquely recognizes influenza B HA. In vivo protection studies in mice confirmed broad cross-reactivity, though limited activity was observed against H2 influenza A and certain influenza B lineages [7,8]. CR9114 achieves this exceptional breadth through a flexible HCDR2 that expands its binding footprint, including contributions from framework region 3 [9].

Plant-based expression systems offer a rapid, scalable, and cost-effective platform for recombinant protein production, including monoclonal antibodies. While mammalian systems typically achieve higher titers (2.0–8.0 g/L), the plant platform provides a faster 5–7 day production cycle with yields of 0.5–1.0 g/kg, significantly reducing capital expenditure and raw material costs [10]. The system provides eukaryotic post-translational modifications that support proper protein folding and functional assembly. Previous studies have demonstrated that plant-produced antibodies against viral pathogens, such as anti-flavivirus monoclonal antibodies (mAbs), exhibited comparable neutralizing potency across all four dengue serotypes relative to mammalian-produced counterparts [11]. Moreover, glycoengineered *Nicotiana benthamiana* lines enable customizable glycosylation profiles, either high-mannose or ΔXF forms, to mimic mammalian-type glycans without plant-specific residues. Notably, the anti-PD-1 monoclonal antibody Pembrolizumab expressed in *N. benthamiana* showed tunable glycoforms that improved FcRn receptor binding and prolonged antibody half-life, thereby enhancing ADCC function [12].

In this study, we aimed to produce the broadly neutralizing antibody CR9114 using the plant expression system. The recombinant antibody was successfully expressed in *Nicotiana benthamiana* and purified via protein A affinity chromatography, followed by protein characterization. We further evaluated its HA binding activity and in vitro neutralization capacity against representative avian influenza A strains, namely A/Jiangsu/NJ210/2023 (H5N1) and A/Gansu/23277/2019 (H7N9). These findings support the potential of plant-derived CR9114 as a universal influenza therapeutic candidate, highlighting the scalability and speed of plant-based production as an alternative platform for pandemic preparedness beyond annually updated seasonal vaccines.

## 2. Materials and Methods

### 2.1. Gene Construction

The amino acid sequences encoding CR9114 were obtained from the Protein Data Bank (PDB ID 4FQY) and were codon optimized for transient expression in *Nicotiana benthamiana* using GeneArt gene synthesis software (Thermo Fisher Scientific, Waltham, MA, USA; web-based proprietary tool with no publicly available version number). Variable regions of the CR9114 light chain (LC) and heavy chain (HC) that were added individually to the human IgG1 kappa (Genbank accession number: AAA58989.1) and gamma chains (Genbank accession number: AAA02914.1) were connected with the SEKDEL ER retention sequence at their C-termini. Synthesized CR9114-HC and CR9114-LC were then cloned into the geminiviral vector pBYR2eK2Md (pBYR2e). The constructed plant expression vectors were transformed into Escherichia coli DH10B by heat shock. Colony polymerase chain reaction (PCR) was used for identification of colonies containing the gene of interest, and these constructs were further checked by sequencing. Confirmed clones were then cultured in Luria–Bertani (LB) media (HiMedia Laboratories, Mumbai, India) containing 50 μg/mL kanamycin (Appli-Chem, Dermstadt, Germany) overnight at 37 °C and the plasmids were isolated and transformed into Agrobacterium tumefaciens GV3101 by electroporation.

### 2.2. Extraction and Purification

Infiltrated leaves were harvested at 4 dpi and homogenized in 1× phosphate-buffered saline (PBS; 137 mM NaCl, 2.7 mM KCl, 4.3 mM NaH_2_PO_4_, and 1.47 mM KH_2_PO_4_, pH 9) using a blender. The homogenate was filtered through Miracloth and subsequently centrifuged at 20,000× *g* for 20 min at 4 °C to remove plant cell debris. The resulting supernatant was passed through a 0.45-μm membrane filter, and the recombinant antibody was purified using a protein A affinity chromatography column (Bestchrom Biosciences, Shanghai, China). Bound proteins were eluted with 100 mM citrate buffer, pH 3, and immediately neutralized with 1.5 M Tris buffer, pH 8.8. The eluted protein was dialyzed against 1× PBS (cell culture grade) and concentrated using an Amicon Ultra centrifugal filter unit with a 50 kDa molecular weight cutoff (MWCO) (Merck Millipore, Burlington, MA, USA). Protein concentration was determined via the Bradford assay following the manufacturer’s protocol (Bio-Rad, Hercules, CA, USA), after which the solution was filtered through a 0.22-μm syringe filter under sterile conditions and stored at −80 °C until further analysis.

### 2.3. Characterization of Plant-Produced CR9114 Antibody

Concentrated protein (2 μg) was mixed with non-reducing loading dye and subjected to SDS-PAGE on either 6% or gradient polyacrylamide gels. Gels were stained with Coomassie Brilliant Blue, and proteins were subsequently transferred onto nitrocellulose membranes using a Mini Trans-Blot system (Bio-Rad). Membranes were blocked with 5% skim milk for 30 min and probed with HRP-conjugated goat anti-human IgG1 gamma chain (1:5000) or goat anti-human IgG1 kappa chain (1:5000) antibodies for 2 h. After thorough washing with 1× PBS, the bound antibodies were visualized using an enhanced chemiluminescence (ECL) substrate.

### 2.4. LC–MS Intact Mass and Peptide Mapping Analysis

Intact mass analysis and peptide mapping were performed by liquid chromatography-mass spectrophotometry (LC–MS) to confirm the molecular mass, sequence integrity, and post-translational modifications of the purified protein, following previously established methods [13]. For intact mass analysis, approximately 20 µg of protein was dissolved in 50 mM ammonium bicarbonate buffer, reduced with 10 mM dithiothreitol (DTT) at 65 °C for 30 min, and centrifuged at 14,000× *g* for 10 min prior to analysis. For peptide mapping, approximately 60 µg of protein was reduced with 10 mM DTT at 65 °C for 30 min, alkylated with 25 mM iodoacetamide (IAA) in the dark at room temperature for 20 min, and digested with trypsin (0.6 µg) at 37 °C for 4 h. The digestion was quenched with 1% formic acid, followed by centrifugation at 14,000× *g* for 10 min.

LC–MS analyses were performed using an Agilent 1290 Infinity II liquid chromatography system coupled to an Agilent 6545XT Q-TOF mass spectrometer. Intact mass separation was carried out on a PLRP-S column, and peptide mapping was performed using an AdvanceBio Peptide Mapping column. All LC and MS parameters were set according to previously reported conditions [13].

### 2.5. Binding Assay

Binding activity of the plant-produced CR9114 antibody to commercial HA antigen was evaluated by ELISA. A 96-well microtiter plate was coated overnight with 2 μg/mL of commercial influenza A HA antigen of A/Vietnam/1194/2004(H5N1) (AcroBiosystem, Newark, DE, USA). Each well of the plate was washed with PBS containing 0.05% Tween-20 (PBS-T) and blocked with 3% skim milk for 2 h. Serial dilutions of the plant-produced CR9114 antibody were then added to the wells and incubated at 37 °C for 2 h, followed by detection using an HRP-conjugated goat anti-human IgG1 gamma chain antibody (1:2000). Enzymatic activity was assessed with 3,3′,5,5′-tetramethylbenzidine (TMB) substrate, and the reaction was stopped with 1 M H_2_SO_4_. Absorbance was measured at 450 nm using an EnSight™ multimode plate reader (PerkinElmer, Springfield, IL, USA), operated using the manufacturer’s default acquisition software (no user-accessible software version). All samples were assayed in duplicate. Binding curves were fitted using a four-parameter logistic (4PL) nonlinear regression model in GraphPad Prism. Kd values were calculated from HA-binding ELISA data based on equilibrium binding analysis.

### 2.6. Cell Lines

Human embryonic kidney (HEK) 293T cells were acquired from ATCC (CRL-3216). HEK293T cells were propagated in Dulbecco’s modified Eagle medium (DMEM) supplemented with 10% fetal bovine serum (FBS). Madin-Darby canine kidney (MDCK) cells were propagated in Opti-MEM (Thermo Fisher Scientific) supplemented with 10% FBS. All cells were maintained in a humidified 37 °C incubator at 5% CO_2_.

### 2.7. Virus Reverse Genetics

The HA and NA genes of A/Jiangsu/NJ210/2023 (H5N1) (GISAID accession number EPI_ISL_17075747) and A/Gansu/23277/2019 (H7N9) (GISAID accession number EPI_ISL_353997) were codon-optimized and synthesized, with the HA designed without the polybasic cleavage site (KRRK in the H5 HA and KRK in the H7 HA). The genes were subsequently cloned into the bi-directional pHW2000 plasmid (a kind gift from Dr. Robert G. Webster, St. Jude Children’s Research Hospital, Memphis, TN, USA [14]) and verified by Sanger sequencing. To perform reverse genetics, HEK293T and MDCK cells were co-cultured in 6-well plates overnight. Cells were then washed with PBS, and the medium was replenished with OptiMEM supplemented with 1 µg/mL TPCK-treated trypsin. pHW2000 plasmids encoding the H5N1 or H7N9 HA and NA were co-transfected along with pHW2000 plasmids encoding the A/Puerto Rico/8/34 (H1N1) PB2, PB1, PA, NP, M and NS genes at 500 ng per plasmid using Fugene HD (Promega). After 2 (H5N1) or 3 (H7N9) days post-transfection, cell-free supernatants were harvested and used to generate further virus stocks in MDCK cells.

### 2.8. Virus Titration

Viruses were quantified by plaque titration. MDCK cells were seeded in 6-well plates overnight in OptiMEM supplemented with 10% FBS and subsequently washed with PBS before use. Ten-fold serial dilutions were performed on the virus stocks using OptiMEM without FBS, and 1 mL of diluted virus was added to the cells. After adsorption for 1 h, cells were washed again with PBS before being overlaid with 0.9% American Bacteriological Agar (Condalab, Madrid, Spain) in minimum essential medium (MEM) supplemented with 1 μg/mL TPCK-treated trypsin. After incubation for 48–72 h at 37 °C, the agar was removed and the cells fixed with 0.1% crystal violet in formaldehyde. To calculate viral titers, the number of plaques in the well containing 10–100 plaques was multiplied by the dilution factor.

### 2.9. Virus Neutralization Assay

MDCK cells were seeded in 96-well plates overnight. The CR9114 antibody was diluted 1:20, and then serial 2-fold dilutions were performed to 1:2560. Each antibody dilution was mixed with 100 plaque-forming units (pfu) of recombinant H5N1 and H7N9 virus prior to addition to the MDCK cells. At 72 h post-treatment, cells were fixed with 80% ice-cold acetone and washed 3 times with PBS-T. Wells were blocked with 2% bovine serum albumin (BSA) in PBS-T for 30 min. Cells were probed with a rabbit anti-influenza A nucleoprotein primary antibody (1:10,000) at room temperature for 1 h followed by an HRP-conjugated goat anti-rabbit IgG antibody (1:10,000) at room temperature for 1 h. Enzymatic activity was assessed using TMB substrate, and the reaction was stopped with 0.5 M H_2_SO_4_. Absorbance was measured at 450 nm (signal) and 620 nm (background) using a microplate reader. All samples were assayed in duplicate. Data was subsequently analyzed using Graphpad Prism. Neutralization curves were fitted by nonlinear least-squares regression to a two-parameter logistic inhibitory model using log10-transformed dilution values. Data were normalized to 0% (virus-only control) and 100% (cell-only control), and NT_50_ values were calculated from the fitted curves with the Hill slope constrained to be negative [15].

### 2.10. Statistical Analysis

All experiments were performed in triplicate. Statistical analyses were conducted using GraphPad Prism 8.0 (GraphPad Software, San Diego, CA, USA). Differences between groups were evaluated using an independent *t*-test. A *p*-value ≤ 0.05 was considered statistically significant.

## 3. Results

### 3.1. Optimization Conditions for Plant-Produced CR9114 Antibody Expression

**Figure 1:**
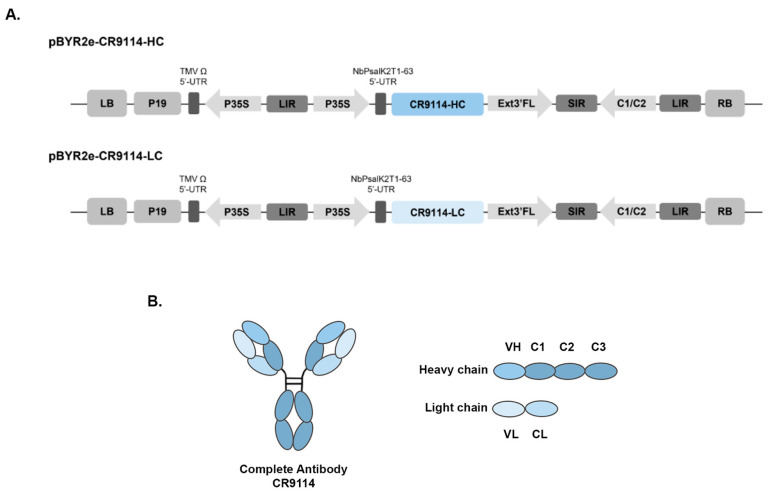
(**A**) Structure of the T-DNA regions of geminiviral vector (pBYR2e) used for expression of the plant-produced CR9114 antibody. P19: P19 silencing suppressor from Tomato Bushy Stunt Virus (TBSV), TMVΩ 5′-UTR: 5′ untranslated region (UTR) of Tobacco Mosaic Virus Ω, P35S: 35S promoter from Cauliflower Mosaic Virus (CaMV), NbP 5′: 5′ UTR of Nicotiana photosystem I reaction center subunit psa K, CR9114 LC: light chain of CR9114 gene, CR9114-HC: heavy chain of CR9114 gene, Ext3′FL: expressed sequence tags- 3′ full length of tobacco extension gene, C2/C1: Bean Yellow Dwarf Virus (BeYDV) ORFs C1 and C2 which encode the replication initiation protein (Rep) and RepA, LIR: long intergenic region of the BeYDV genome, SIR: short intergenic region of the BeYDV genome, LB and RB: the left and right borders of the Agrobacterium T-DNA region. (**B**) Illustration of the complete CR9114 antibody with its constant and variable regions.

**Figure 2:**
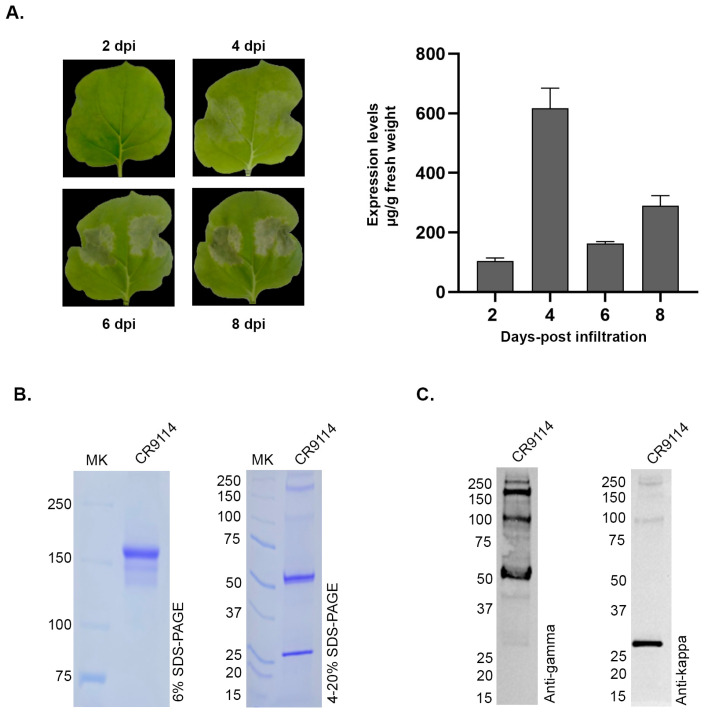
(**A**) Day-post-infiltration (dpi) optimization conditions of *Nicotiana benthamiana* leaves at 2, 4, 6, and 8 days post-infiltration (dpi). Expression levels were quantified by ELISA and presented as µg per g fresh weight. (**B**) SDS-PAGE of purified CR9114 antibody in a non-reducing 6% gel and a reducing gradient 4–20% gel. (**C**) Western blot detection using anti-gamma and anti-kappa antibodies.

The CR9114 antibody was engineered for plant production by fusing its variable regions to a human IgG1 constant region carrying a C-terminal ER-retention signal (SEKDEL) (Figure 1A). After synthesis and codon optimization for *N. benthamiana*, each chain was inserted into the pBYR2e vector and introduced into A. tumefaciens GV3101 for transient expression. Heavy- and light-chain cultures were mixed for co-infiltration, allowing the antibody to assemble inside the plant cells. To determine the optimal harvest time, infiltrated leaves were collected at 2, 4, 6, and 8 days post-infiltration (dpi). Mild necrosis was first visible at 4 dpi, becoming more pronounced by 6 dpi (Figure 2A). Antibody accumulation was quantified by ELISA, using polyclonal human IgG as the capture antibody and anti-gamma HRP for detection. CR9114 expression increased after infiltration and reached its highest level among the tested time points at approximately 600 µg/g fresh weight on day 4, followed by a decline at 6 dpi and a slight increase again by 8 dpi (Figure 2A).

### 3.2. Protein Purification of CR9114 Produced in Nicotiana benthamiana

Antibody purification was carried out with *N. benthamiana* leaves co-infiltrated with CR9114 heavy and light chains. Leaves were harvested at 4 dpi, corresponding to the highest expression levels observed among the tested time points, and crude extracts were subjected to protein A affinity chromatography. The purified antibody was buffer-exchanged and concentrated using an Amicon ultrafiltration membrane. The concentration of the purified protein was determined by Bradford assay. The standard curve, generated using BSA standards, demonstrated a linear range of 0–800 μg/mL with a correlation coefficient (R^2^) of 0.9899. Analysis by SDS-PAGE and Western blot confirmed the integrity of the purified CR9114. Under non-reducing conditions, a single band at ~150 kDa indicated fully assembled IgG, while reducing conditions resolved the antibody into the expected ~50 kDa heavy chain and ~25 kDa light chain. Western blotting further verified the identity of each chain, with anti-gamma antibodies detecting the heavy chain and anti-kappa antibodies recognizing the light chain, demonstrating correct assembly of the plant-produced antibody (Figure 2B).

Molecular integrity of the purified CR9114 antibody was further confirmed by LC–MS intact mass analysis. The observed intact molecular mass of 149,654.2 Da was higher than the theoretical mass of the non-glycosylated antibody (146,429.2 Da), consistent with the presence of high-mannose N-glycans commonly associated with antibodies expressed in *N. benthamiana*. In addition, LC–MS peptide mapping provided approximately 55–60% sequence coverage, meeting basic identity confirmation criteria, with detailed peptide coverage presented in the Supplementary Figure S2.

### 3.3. Binding with H5N1 Influenza Virus

**Figure 3:**
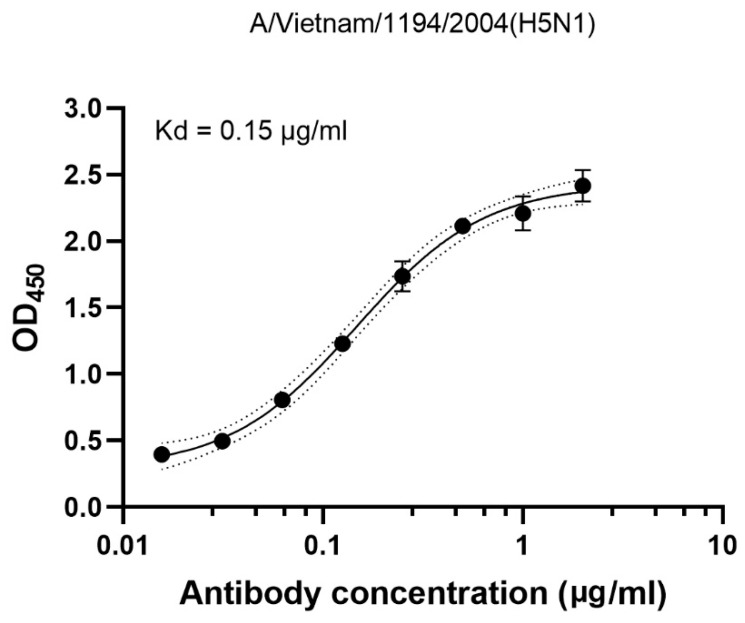
Binding of CR9114 to the HA antigen of A/Vietnam/1194/2004 (H5N1) by ELISA.

Binding activity of the plant-produced CR9114 antibody was examined by indirect ELISA using 2 μg/mL of the commercial HA antigen from A/Vietnam/1194/2004 (H5N1) as the capture antigen. All samples were assayed in duplicate. As shown in Figure 3, the plant-produced CR9114 antibody exhibited a calculated equilibrium dissociation constant (Kd) of 0.15 µg/mL, which corresponds to a high-affinity binding value of 1.00 nM (calculated using a molecular weight of 150 Kd). These results demonstrate that the plant-produced CR9114 antibody effectively recognizes and binds H5N1 HA.

### 3.4. Neutralization of Influenza Virus

To demonstrate that the plant-produced CR9114 antibody retains neutralization activity against influenza viruses, live virus neutralization was tested using recombinant A/Jiangsu/NJ210/2023 (H5N1) and A/Gansu/23277/2019 (H7N9) in the attenuated A/Puerto Rico/8/34 (H1N1) strain backbone. These two strains were selected as recent highly pathogenic avian influenza A viruses that were successfully transmitted to human hosts and subsequently caused host mortality. These viruses were rescued by reverse genetics to carry the HA and NA of the H5N1 or H7N9 virus, but the remaining genes (PB2, PB1, PA, NP, M, NS) were derived from the attenuated strain (Supplementary Figure S3A) and titered by plaque assay (Supplementary Figure S3B). The recombinant viruses were incubated with increasing dilutions of the antibody and used to infect MDCK cells.

**Figure 4:**
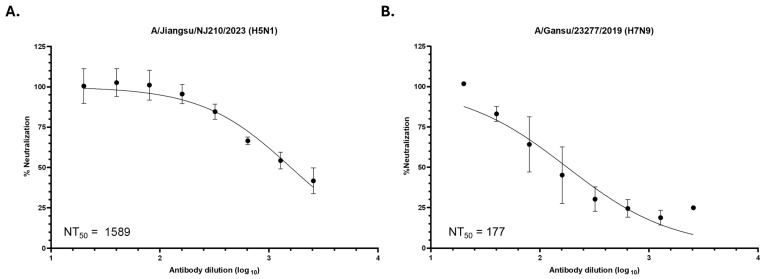
Neutralization of recombinant H5N1 and H7N9 by CR9114. (**A**) Recombinant A/Jiangsu/NJ210/2023 (H5N1) and (**B**) A/Gansu/23277/2019 (H7N9) were incubated with increasing dilutions of CR9114 prior to addition to MDCK cells. After 3 days, cells were fixed and probed for influenza A nucleoprotein (NP) expression.

Strong neutralization of the H5N1 virus by CR9114 was observed with a 50% neutralization titer (NT_50_) of 1589, which is equivalent to an IC_50_ of 200 ng/mL or 1.33 nM (Figure 4A). Neutralization was also seen against the H7N9 virus, with an NT_50_ of 177 or an IC_50_ of 1.79 µg/mL (11.93 nM) (Figure 4B). These results demonstrate that CR9114 is capable of neutralizing genetically divergent avian influenza strains recently isolated from humans despite being based on a 2004 H5N1 virus.

## 4. Discussion

Our study demonstrates that plant-produced CR9114, a broadly neutralizing monoclonal antibody targeting the conserved hemagglutinin (HA) stem, retains functional neutralizing activity against recently isolated avian influenza viruses. The antibody exhibited strong neutralization against A/Jiangsu/NJ210/2023 (H5N1; IC_50_ ≈ 200 ng/mL) and weaker but still detectable activity against A/Gansu/23277/2019 (H7N9; IC_50_ = 1.79 µg/mL). Although a direct comparison with mammalian cell–produced CR9114 was not performed in this study, the neutralization potency observed here falls within the range previously reported for CR9114 expressed in mammalian systems. Earlier studies demonstrated that CR9114 potently neutralizes H5N1 viruses across multiple clades (0, 1, 2.1.3.2, 2.2, and 2.3.4.4), with reported IC_50_ values ranging from 0.646 to 86.6 ng/mL in pseudotyped virus assays [16]. In contrast, reduced activity has been reported against certain Group 2 HA viruses, such as H3N2, with IC_50_ values around 6.25 µg/mL [17]. These previously reported trends are consistent with our findings, in which stronger activity was observed against the Group 1 H5N1 strain and comparatively lower activity against the Group 2 H7N9 strain. Overall, the neutralization profile of plant-produced CR9114 aligns with the established breadth and subtype-dependent potency described for this antibody.

The use of recombinant influenza viruses for assessing antibody-mediated neutralization in vitro enables safer assessment of highly pathogenic avian influenza viruses outside high-containment settings. By using only the HA and NA genes of influenza viruses of interest, neutralization activity becomes a direct function of antibody-mediated inhibition of virus attachment and membrane fusion, eliminating the confounding effects arising from strain-specific internal genes and their interaction with host cellular machinery. Nevertheless, these recombinant viruses possibly differ from the original viruses in HA density and HA-NA stoichiometry, which can in turn affect the effectiveness of antibody neutralization and therefore assay sensitivity [18]. Despite these differences, recombinant viruses have shown strong correlation in neutralization assays compared to the original virus [19]. Finally, if multi-round inhibition were to be assessed using longer incubation times, the differences in replication kinetics between PR8 and the original viruses would increasingly impact the relevance of the data.

The reduced neutralization potency observed against H7N9 highlights a well-characterized structural limitation of stem-directed antibodies when encountering Group 2 influenza A viruses. Although site-specific glycosylation of the HA protein from A/Gansu/23277/2019 (H7N9) was not experimentally characterized in this study, prior structural analyses have demonstrated that an N-linked glycan at Asn38 (HA2) in Group 2 hemagglutinins can sterically hinder binding of stem-directed antibodies such as CR9114. Crystallographic studies have shown that this glycan partially occludes the conserved stem epitope, requiring CR9114 to undergo a relative rotation of its VH domain and to rely on the conformational flexibility of its HCDR3 loop to maintain binding [7]. This structural constraint is absent in Group 1 viruses such as H5N1, which instead contain a non-glycosylated His38 residue at the corresponding position. Consistent with this mechanism, previous studies have shown that the stem-directed antibody CR6261 exhibits reduced neutralization against A/Anhui/1/2013 (H7N9), a strain bearing the Asn38 glycosylation site, supporting the role of this glycan in limiting stem-directed antibody activity against Group 2 influenza viruses [20]. Together, our in vitro findings are consistent with the established structural and mechanistic understanding of CR9114–HA interactions in Group 2 influenza viruses.

The protective efficacy of HA-stem-targeted antibody CR9114 has been attributed to Fc-mediated effector functions. Previous in vivo studies demonstrated that even when CR9114 fails to neutralize a virus in traditional microneutralization assays, it can still confer 100% protection in lethal mouse models via antibody-dependent cellular cytotoxicity (ADCC) [7]. This was further substantiated by the use of “LALA” mutant variants, which abolish Fc-receptor binding and significantly diminish the antibody’s protective capacity, suggesting that the recruitment of immune effector cells to the HA stem is critical when fusion inhibition is sterically hindered by glycans [8].

However, recent shifts in administration strategies suggest that the requirement for Fc-effector functions may be route-dependent. Airway delivery (intranasal or nebulization) of stem-binding antibodies has been shown to be 10- to 50-fold more effective than systemic routes for treating various influenza strains [21]. Specifically, Beukenhorst et al. demonstrated that intranasal administration of CR9114 provides robust prophylactic protection by neutralizing the virus directly at the respiratory entry point [16]. Notably, recent Phase 1 and preclinical findings have introduced a critical nuance: while systemic protection is heavily Fc-dependent, intranasal delivery may operate through an Fc-independent mechanism [22,23]. This suggests that in the mucosal environment, direct neutralization or viral exclusion (a “molecular mask” effect) may be the primary driver of efficacy. While this highlights a currently inconclusive area regarding the necessity of Fc-recruitment in different anatomical compartments, it underscores the immense clinical potential of CR9114, which is currently progressing through human trials.

CR9114 is widely used within the influenza research community as a benchmark broadly neutralizing antibody, serving as an essential positive control and comparator for novel stem-directed candidates [17,24]. While recombinant expression in mammalian cell lines often relies on serum-dependent media, leading to potential contamination with bovine IgGs, the plant-based expression system (*Nicotiana benthamiana*) offers a rapid, scalable, and serum-free alternative. Our platform facilitates this transition by enabling the rapid production of CR9114 in dimeric or polymeric IgA formats. Given the advantages of IgA in the upper respiratory tract [25] and the specific restriction of IgA Fc-effector functions to stem-targeting antibodies [26], this isotype switch could significantly enhance mucosal stability and localized neutralization compared to standard IgG.

In addition, a significant advantage of the plant system is the ability to produce “tailor-made” glycosylation. It is well-established that specific glycan profiles modulate the indirect protective activity of antibodies via modulating effector functions such as ADCC and phagocytosis. For example, removal of core fucose enhances FcγRIIIa binding and increases ADCC activity, while optimized glycan structures can also improve serum persistence and functional stability. Recent reports demonstrated the ΔXT glycoengineered plants can eliminate plant-specific glycans and produce human-compatible N-glycan profiles, resulting in improved Fc receptor engagement and enhanced effector functions [27,28]. Consistently, our previous study on glycoengineered plant-produced pembrolizumab showed prolonged serum half-life and enhanced Fc-mediated activity compared with the wild-type plant-produced antibody [12]. These findings indicate that targeted glycoengineering or isotype optimization of plant-produced CR9114 could further improve its neutralizing activity and Fc-mediated effector functions, thereby strengthening its potential as an effective “molecular mask” prophylactic strategy.

## 5. Conclusions

In summary, this study demonstrates that *Nicotiana benthamiana* is a robust and scalable platform for the production of the broadly neutralizing antibody CR9114. Our results confirm that plant-derived CR9114 maintains its structural integrity and epitope specificity, preserving critical neutralizing activity against diverse influenza strains, including contemporary H5N1 avian influenza threats. Furthermore, the compatibility of this system with isotype switching and glycoengineering provides a pathway for the development of “molecular mask” prophylactics for intranasal administration. Ultimately, these findings solidify the role of plant-expressed antibodies in the global toolkit for pandemic influenza preparedness and proactive mucosal defense.

## Data Availability

The original contributions presented in this study are included in the article/Supplementary Material. Further inquiries can be directed to the corresponding author.

## Supplementary Materials

The following supporting information can be downloaded at: https://www.mdpi.com/article/10.3390/vaccines14030222/s1, Figure S1: (A) A Bradford standard curve, (B) Raw SDS PAGE and Western blot film; Figure S2: Protein mass using LC/MS. (A) Intact mass, (B) subunit mass; light chain, (C) subunit mass; heavy chain; Figure S3: (A) Recombinant viruses A/Jiangsu/NJ210/2023 (H5N1) or A/Gansu/23277/2019 (H7N9), (B) virus titers.

## References

[1] World Health Organization (WHO) (2025). Influenza (Seasonal). https://www.who.int/news-room/fact-sheets/detail/influenza-(seasonal).

[2] Molinari N.-A.M., Ortega-Sanchez I.R., Messonnier M.L., Thompson W.W., Wortley P.M., Weintraub E., Bridges C.B. (2007). The annual impact of seasonal influenza in the US: Measuring disease burden and costs. Vaccine.

[3] Taylor L. (2025). First ever human case of H5N5 avian flu confirmed in the US. BMJ.

[4] World Health Organization (WHO) (2025). Disease Outbreak News; Avian Influenza A (H5N1) in Cambodia. https://www.who.int/emergencies/disease-outbreak-news/item/2024-DON501.

[5] Laursen N.S., Wilson I.A. (2013). Broadly neutralizing antibodies against influenza viruses. Antivir. Res..

[6] Corti D., Voss J., Gamblin S.J., Codoni G., Macagno A., Jarrossay D., Vachieri S.G., Pinna D., Minola A., Vanzetta F. (2011). A neutralizing antibody selected from plasma cells that binds to group 1 and group 2 influenza A hemagglutinins. Science.

[7] Dreyfus C., Laursen N.S., Kwaks T., Zuijdgeest D., Khayat R., Ekiert D.C., Lee J.H., Metlagel Z., Bujny M.V., Jongeneelen M. (2012). Highly conserved protective epitopes on influenza B viruses. Science.

[8] Sutton T.C., Lamirande E.W., Bock K.W., Moore I.N., Koudstaal W., Rehman M., Weverling G.J., Goudsmit J., Subbarao K. (2017). In Vitro Neutralization Is Not Predictive of Prophylactic Efficacy of Broadly Neutralizing Monoclonal Antibodies CR6261 and CR9114 against Lethal H2 Influenza Virus Challenge in Mice. J. Virol..

[9] Beukenhorst A.L., Frallicciardi J., Koch C.M., Klap J.M., Phillips A., Desai M.M., Wichapong K., Nicolaes G.A.F., Koudstaal W., Alter G. (2022). The influenza hemagglutinin stem antibody CR9114: Evidence for a narrow evolutionary path towards universal protection. Front. Virol..

[10] Nandi S., Kwong A.T., Holtz B.R., Erwin R.L., Marcel S., McDonald K.A. (2016). Techno-economic analysis of a transient plant-based platform for monoclonal antibody production. MAbs.

[11] Krittanai S., Rattanapisit K., Bulaon C.J.I., Pitaksajjakul P., Keadsanti S., Ramasoota P., Strasser R., Phoolcharoen W. (2024). Nicotiana benthamiana as a potential source for producing anti-dengue virus D54 neutralizing therapeutic antibody. Biotechnol. Rep..

[12] Bulaon C.J.I., Jaratsittisin J., Rattanapisit K., Suwanchaikasem P., Guo S., Boonha K., Pitaksajjakul P., Simsom N., Limprasutr V., Phoolcharoen W. (2025). Glycoengineering of plant-produced Pembrolizumab enhances FcRn binding and extends serum half-life in mice. Biotechnol. Rep..

[13] Phakham T., Bulaon C.J.I., Khorattanakulchai N., Shanmugaraj B., Buranapraditkun S., Boonkrai C., Sooksai S., Hirankarn N., Abe Y., Strasser R. (2021). Functional Characterization of Pembrolizumab Produced in Nicotiana benthamiana Using a Rapid Transient Expression System. Front. Plant Sci..

[14] Hoffmann E., Neumann G., Kawaoka Y., Hobom G., Webster R.G. (2000). A DNA transfection system for generation of influenza A virus from eight plasmids. Proc. Natl. Acad. Sci. USA.

[15] Ferrara F., Temperton N. (2018). Pseudotype Neutralization Assays: From Laboratory Bench to Data Analysis. Methods Protoc..

[16] Beukenhorst A.L., Frallicciardi J., Rice K.L., Koldijk M.H., de Mello J.C.M., Klap J.M., Hadjichrysanthou C., Koch C.M., da Costa K.A.S., Temperton N. (2024). A pan-influenza monoclonal antibody neutralizes H5 strains and prophylactically protects through intranasal administration. Sci. Rep..

[17] Yasuhara A., Yamayoshi S., Ito M., Kiso M., Yamada S., Kawaoka Y. (2018). Isolation and Characterization of Human Monoclonal Antibodies That Recognize the Influenza A(H1N1)pdm09 Virus Hemagglutinin Receptor-Binding Site and Rarely Yield Escape Mutant Viruses. Front. Microbiol..

[18] Gao J., Wan H., Li X., Martinez M.R., Klenow L., Gao Y., Ye Z., Daniels R. (2021). Balancing the influenza neuraminidase and hemagglutinin responses by exchanging the vaccine virus backbone. PLoS Pathog..

[19] Yang J., Li W., Long Y., Song S., Liu J., Zhang X., Wang X., Jiang S., Liao G. (2014). Reliability of Pseudotyped Influenza Viral Particles in Neutralizing Antibody Detection. PLoS ONE.

[20] Throsby M., van den Brink E., Jongeneelen M., Poon L.L.M., Alard P., Cornelissen L., Bakker A., Cox F., Van Deventer E., Guan Y. (2008). Heterosubtypic Neutralizing Monoclonal Antibodies Cross-Protective against H5N1 and H1N1 Recovered from Human IgM+ Memory B Cells. PLoS ONE.

[21] Vigil A., Frias-Staheli N., Carabeo T., Wittekind M. (2020). Airway Delivery of Anti-influenza Monoclonal Antibodies Results in Enhanced Antiviral Activities and Enables Broad-Coverage Combination Therapies. J. Virol..

[22] Beukenhorst A.L., Rogiers R., Rice K.L., Booth G., Haasnoot J., Nkolola J., Ayala J., Wang L., Julg B., Pastini A.K. (2026). Phase 1 and preclinical studies reveal safety, pharmacokinetics, and efficacy of intranasal delivery of the influenza antibody CR9114. Sci. Transl. Med..

[23] Beukenhorst A.L., Rice K.L., Frallicciardi J., Koldijk M.H., Boudreau C.M., Crawford J., Cornelissen L.A., da Costa K.A., de Jong B.A., Fischinger S. (2025). Intranasal administration of a panreactive influenza antibody reveals Fc-independent mode of protection. Sci. Rep..

[24] Dunand C.J.H., Leon P.E., Kaur K., Tan G.S., Zheng N.Y., Andrews S., Huang M., Qu X., Huang Y., Salgado-Ferrer M. (2015). Preexisting human antibodies neutralize recently emerged H7N9 influenza strains. J. Clin. Investig..

[25] Biswas M., Yamazaki T., Chiba J., Akashi-Takamura S. (2020). Broadly Neutralizing Antibodies for Influenza: Passive Immunotherapy and Intranasal Vaccination. Vaccines.

[26] Freyn A.W., Han J., Guthmiller J.J., Bailey M.J., Neu K., Turner H.L., Rosado V.C., Chromikova V., Huang M., Strohmeier S. (2021). Influenza hemagglutinin-specific IgA Fc-effector functionality is restricted to stalk epitopes. Proc. Natl. Acad. Sci. USA.

[27] Yang M., Sun H., Lai H., Neupane B., Bai F., Steinkellner H., Chen Q. (2023). Plant-Produced Anti-Zika Virus Monoclonal Antibody Glycovariant Exhibits Abrogated Antibody-Dependent Enhancement of Infection. Vaccines.

[28] Nguyen K.D., Kajiura H., Kamiya R., Yoshida T., Misaki R., Fujiyama K. (2023). Production and N-glycan engineering of Varlilumab in Nicotiana benthamiana. Front. Plant Sci..

