# Supplementary material for: Plant-Produced Broadly Neutralizing Influenza Monoclonal Antibody CR9114 Exhibits Activity Against Heterologous Avian Influenza Viruses
Source: Vaccines (Basel). 2026 Feb 28;14(3):222. doi: 10.3390/vaccines14030222 (PMC13030651; doi:10.3390/vaccines14030222)

Supplementary data

Supplementary Figure S1: (A) A Bradford standard curve was generated using Bovine Serum Albumin (BSA) at concentrations ranging from 0 to 800 ug/ml. Absorbance was measured at 595 nm. The linear regression equation is y = (m)x + c with a correlation coefficient (R^2) of 0.9899. (B) Raw SDS-PAGE and western blot film of Figure 2B, C

A.

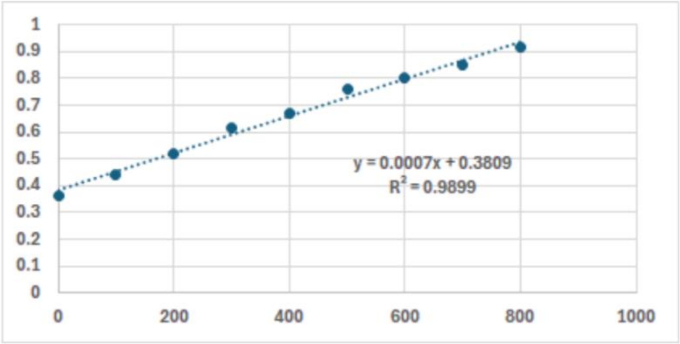

B.

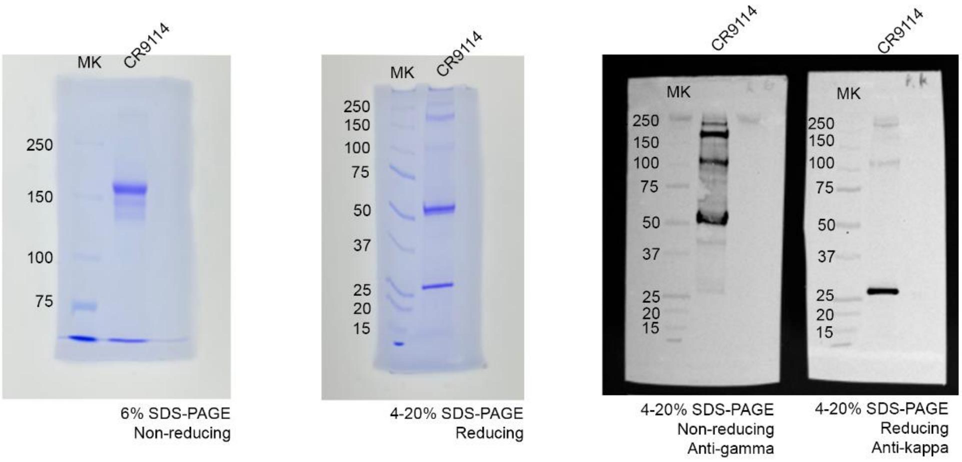

Supplementary Figure S2: Protein mass using LC/MS. (A) Intact mass, (B) subunit mass; light chain, (C) subunit mass; heavy chain. The x-axis represents the deconvoluted molecular mass in atomic mass units (amu). The y-axis represents the relative ion abundance (intensity) measured in counts.

A.

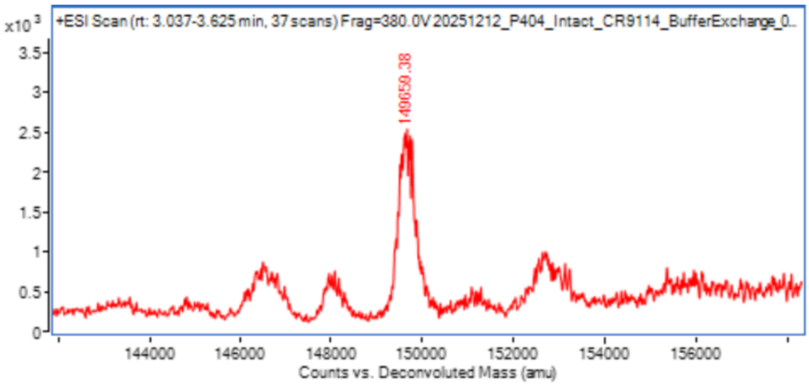

B.

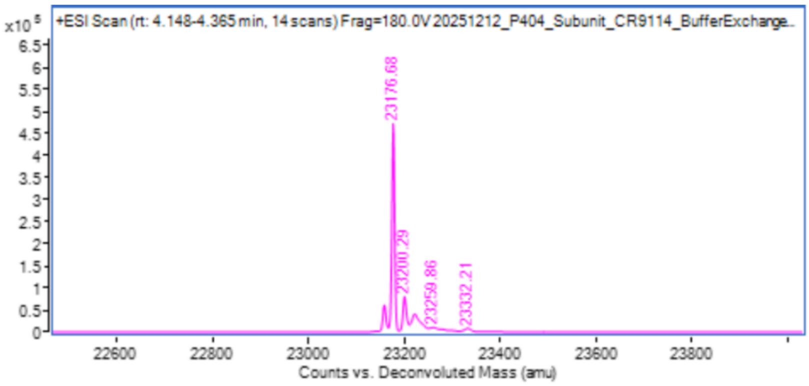

C.

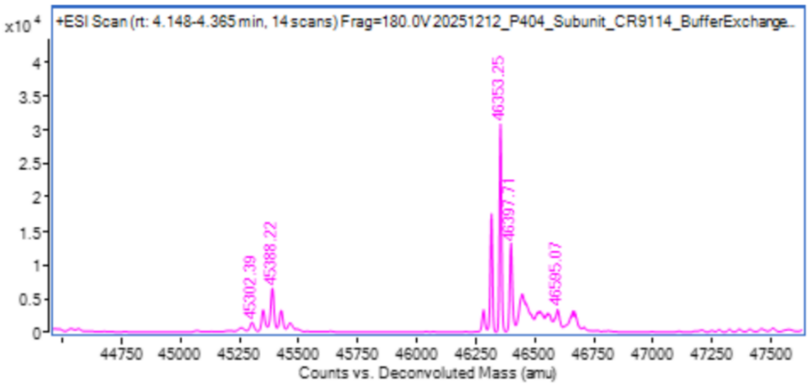

Supplementary Figure S3: Recombinant viruses. (A) Recombinant A/Puerto Rico/8/34 (H1N1) viruses carrying the HA and NA genes of A/Jiangsu/NJ210/2023 (H5N1) or A/Gansu/23277/2019 (H7N9) were generated by reverse genetics. (B) Recombinant viruses were passaged in MDCK cells and virus titers were assessed by plaque titration.

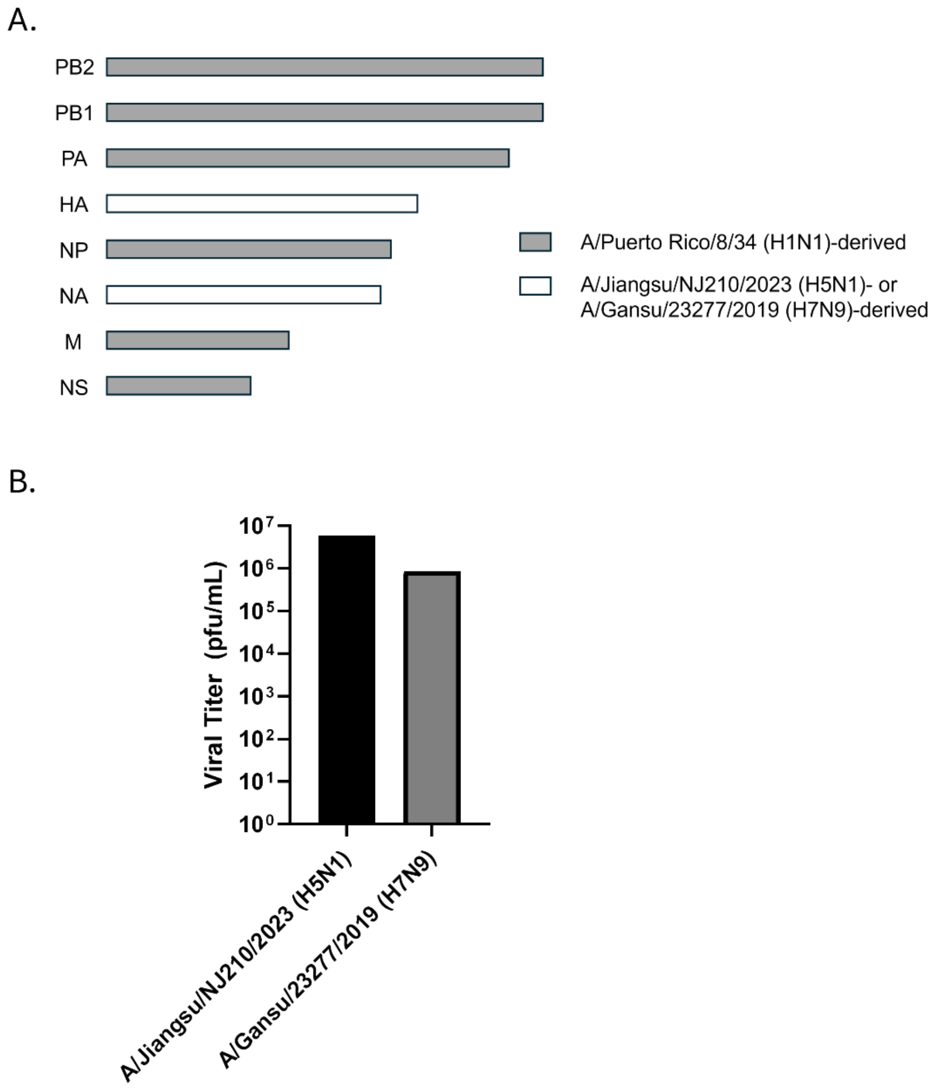

Supplement: Supplementary file 1 [file vaccines-14-00222-s001.zip › vaccines-4155400-supplementary.pdf]
